# Supplementary material for: Contrasting epigenetic control of transgenes and endogenous genes promotes post-transcriptional transgene silencing in Arabidopsis
Source: Nat Commun. 2021 May 13;12:2787. doi: 10.1038/s41467-021-22995-3 (PMC8119426; doi:10.1038/s41467-021-22995-3)
Supplement: Supplementary file 1 — Supplementary Information [file 41467_2021_22995_MOESM1_ESM.pdf]

# Contrasting epigenetic control of transgenes and endogenous genes promotes post-transcriptional transgene silencing in *Arabidopsis*

Butel and Yu *et al.*

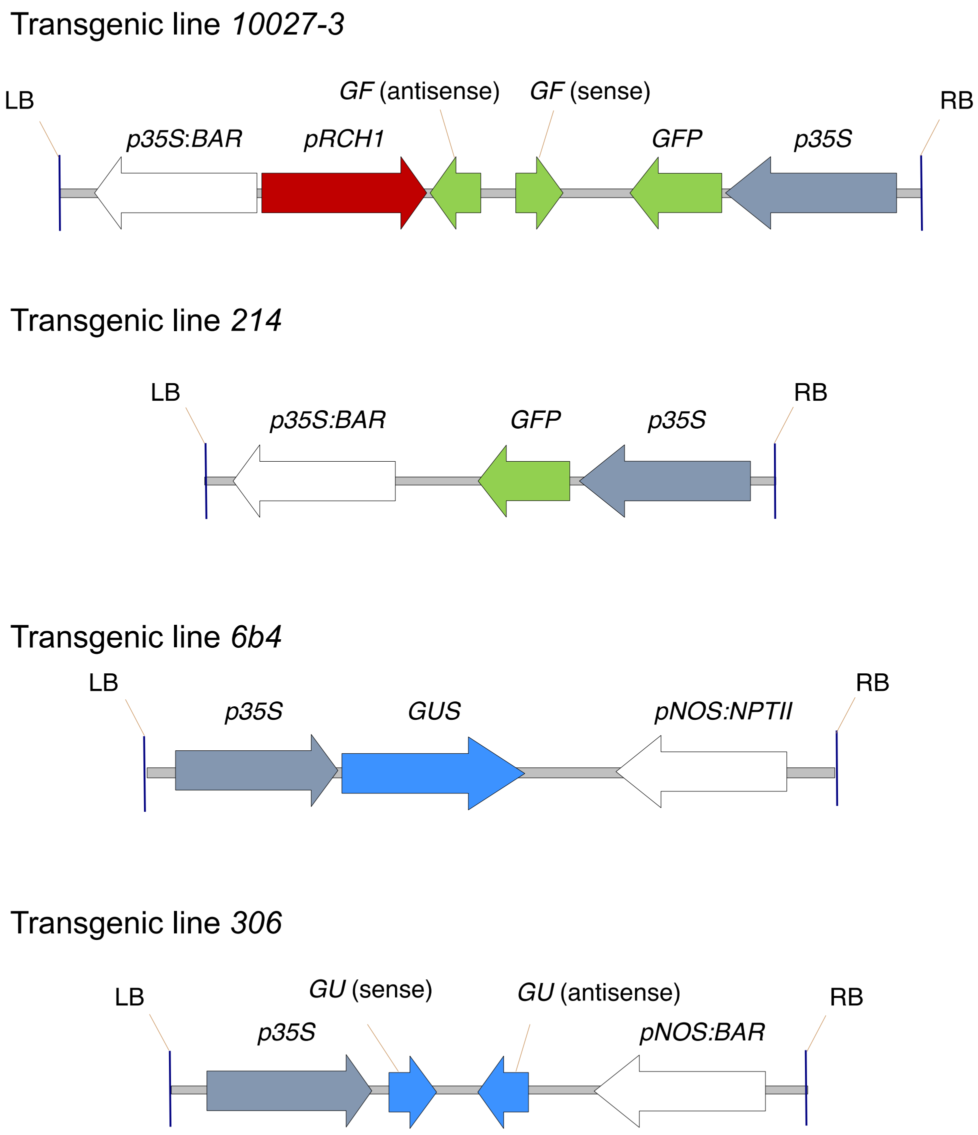


**Supplementary Figure 1. T-DNA maps for transgenic reporter lines of *Arabidopsis*.**
A *BAR* transgene was used as a selectable transformation marker for transgenic lines *10027-3* [1], *214* [2], and *306* [3], whereas a *NPTII* transgene was used as a selectable transformation marker for transgenic line *6b4* [3]. The *pRCH1* promoter drives root tip-specific expression of the *GF* hairpin RNA in transgenic line 10027-3. LB and RB, left- and right-borders of the T-DNA.

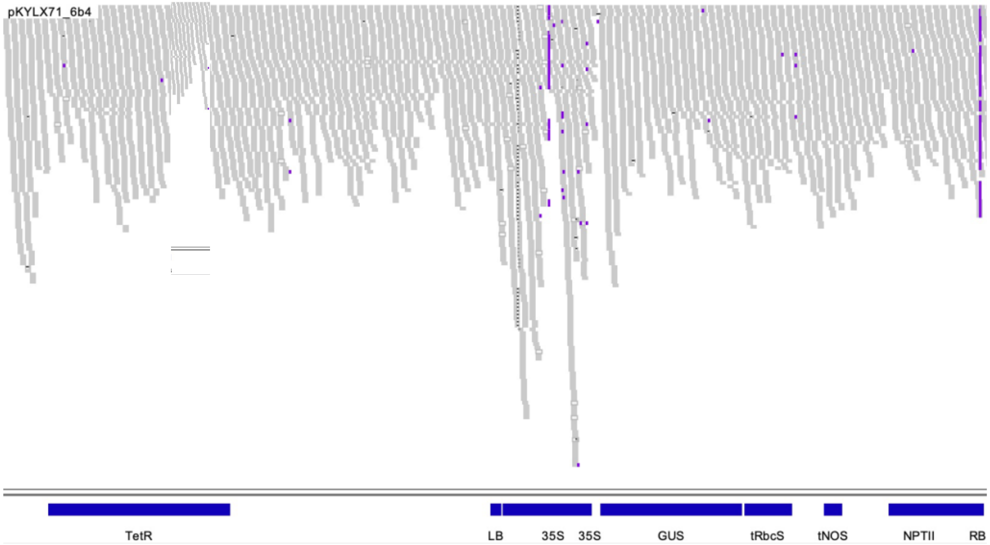


**Supplementary Figure 2. Read coverage reveals integration of the entire plasmid at the *6b4* locus.**
Browser track showing bisulfite paired-end reads mapping to the *6b4* locus. Purple lines represent single nucleotide errors in the reads or reference sequence. The *6b4* locus includes the tetracycline resistance gene (*TetR*), the left- and right-border sequences (LB and RB) flanking the *35S* promoter (*p35S*), *GUS* coding sequence (*GUS*) and *Rubisco* terminator (tRbcS), and the *NOS* promoter (*NOSp*) and terminator (*tNOS*) driving the kanamycin resistance gene (*NPTII*).

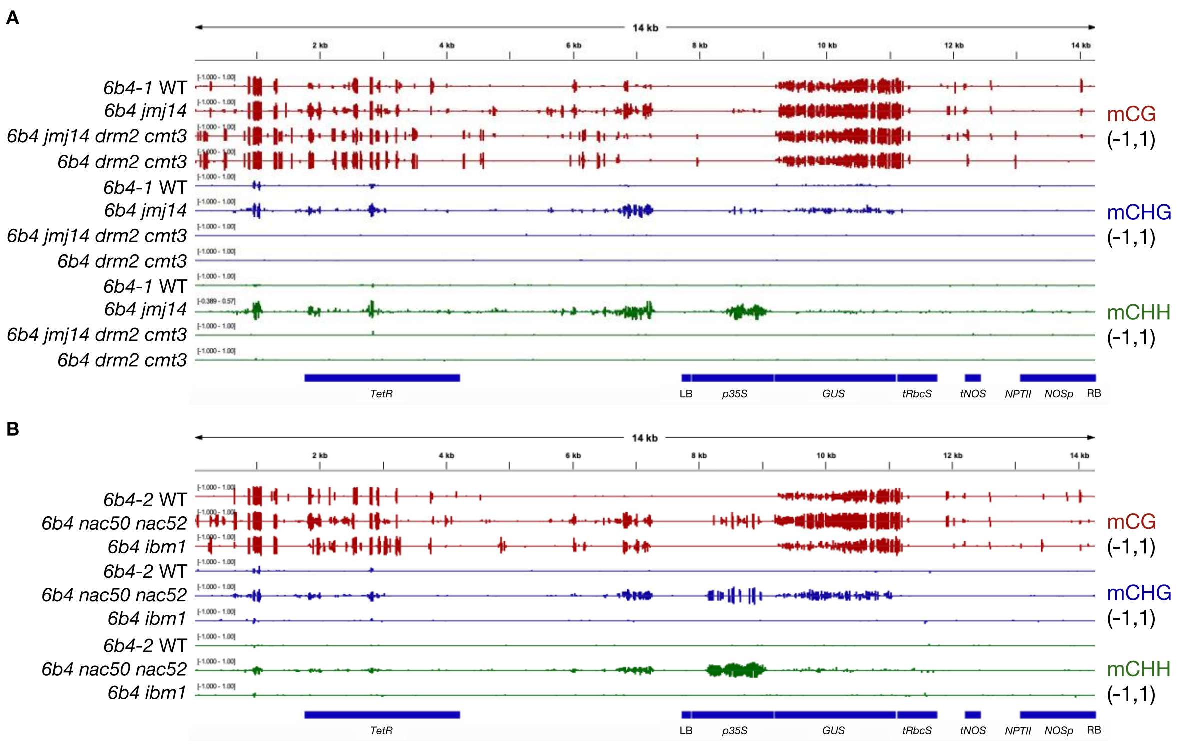


**Supplementary Figure 3. JMJ14 and NAC50/NAC52 prevents CHG and CHH methylation over the majority of the *6b4* locus.**
Browser tracks showing CG, CHG and CHH methylation levels for the entire *6b4* locus in leaf tissue from (A) 5-week- and (B) 8-week-old plants. The *6b4* locus includes the tetracycline resistance gene (*TetR*), the left- and right-border sequences (LB and RB) flanking the *35S* promoter (*p35S*), *GUS* coding sequence (*GUS*) and *Rubisco* terminator (tRbcS), and the *NOS* promoter (*NOSp*) and terminator (*tNOS*) driving the kanamycin resistance gene (*NPTII*). Bisulfite data were analyzed using R version 4.0.2.

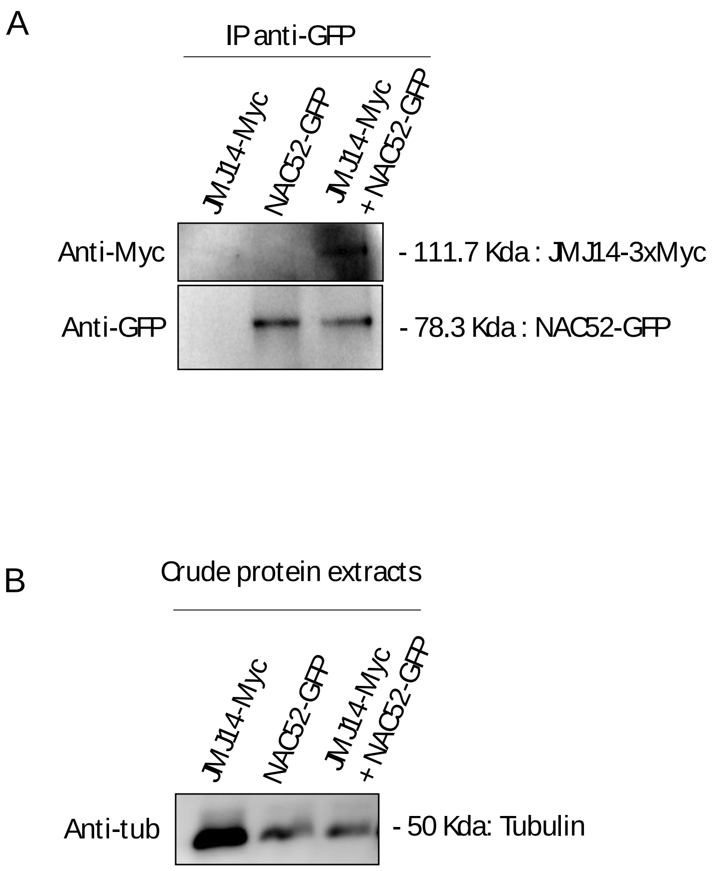


**Supplementary Figure 4. JMJ14 and NAC52 interact.**
Proteins were extracted from plants carrying *pJMJ14:JMJ14-Myc, pUBQ10:NAC52-GFP* or both constructs and submitted to GFP immunoprecipitation. A. Immunoprecipitated proteins were separated on SGS-page gel and analyzed after transfert using anti-Myc and anti-GFP antibodies. B. A distinct gel run with crude extracts was analyzed using anti-tubulin antibodies to ensure that similar amounts of proteins were used for immunoprecipitation. Uncropped and unprocessed scans of images are provided as a Source Data file.

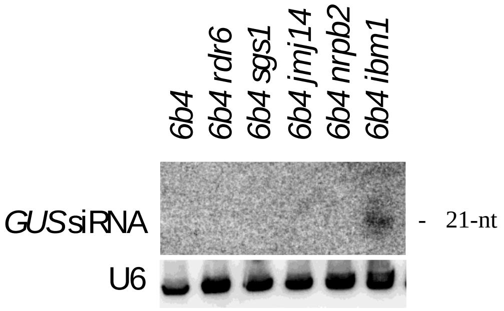


**Supplementary Figure 5. IBM1 impairment provokes spontaneous PTGS of *GUS* at the *6b4* locus.**
LMW RNAs were extracted from bulks of 50 plants of the indicated genotypes grown in vitro for 18 days. The Northen blot was hybridized with a *GUS* probe, revealing the presence of *GUS* siRNAs in *6b4 ibm1* plants, indicating that the *p35S:GUS* transgene is silenced by PTGS in this mutant background. Uncropped and unprocessed scans of images are provided as a Source Data file.

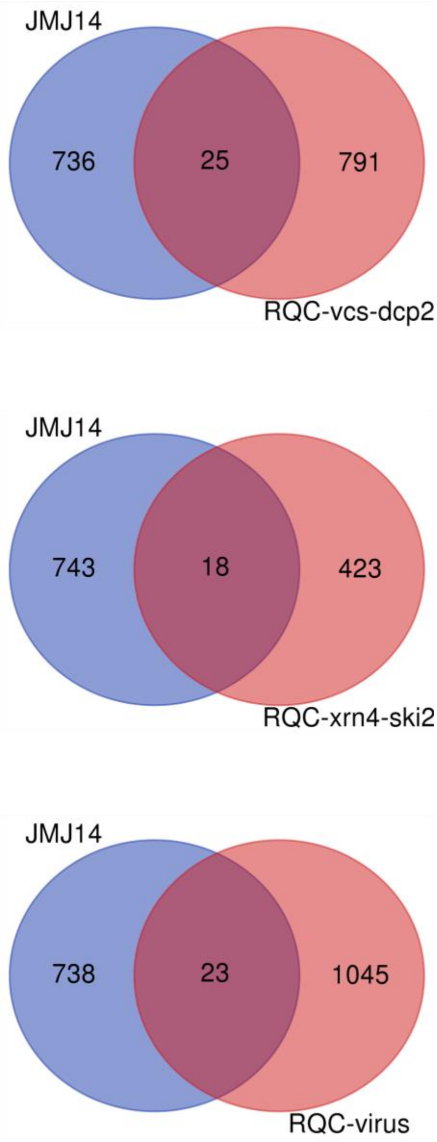


**Supplementary Figure 6. JMJ14 binding is not required for the production of siRNAs from endogenous genes when RQC is impaired.**
Venn diagrams showing the overlap between JMJ14-binding genes and endogenous genes producing siRNAs when RQC is impaired by *dcp2* or *vcs* mutations, *ski2* and *xrn4* mutations or infection by viruses. In each case, the overlap is similar to that expected by chance.

**Supplementary Table 1. List of the primers used in this study.**

| **Primer name** | **Sequence** | **Used for** |
|---|---|---|
| Promoter 35S-F | CTACAAATGCCATCATTGCG | ChIP |
| Promoter 35S-R | AAGGATAGTGGGATTGTGCG | ChIP |
| GUS 5'-F | TCCTGTAGAAACCCCAACCC | ChIP |
| GUS 5'-R | TGGCCTGCCCAACCTTT | ChIP |
| GUS 3'-F | GTATCAGTGTGCATGGCTGG | ChIP |
| GUS 3'-R | AGTTCATGCCAGTCCAGCG | ChIP |
| GAPDH-F | GGTACGACAACGAATGGGGT | ChIP |
| GAPDH-R | TGACTGCGCATGGAATCAGT | ChIP |
| JMJ14 Q183*-F | CCTCTGAAGGAGAAAAAAATATGGGAGAA | *jmj14-5* genotyping |
| JMJ14 Q183*-R | GCATTCCTTAAAGTATTCATCATACTCCT | *jmj14-5* genotyping |
| JMJ14 Intron521-F | TGTGGAATTGTGTTTATTTCCTGCA | *jmj14-6* genotyping |
| JMJ14 Intron521-R | TGATAAGCAAAAGCCACGAACTTAA | *jmj14-6* genotyping |
| JMJ14 G331E-F | TGTGCTTTCTAGGAGCTATGTACTAACC | *jmj14-7* genotyping |
| JMJ14 G331E-R | AGACTCAGCATGGTTTCCAGGGGTC | *jmj14-7* genotyping |

**Supplementary Table 2. Summary of whole-genome bisulfite sequencing.**

| **Sample** | **Median coverage** | **mCG (%)** | **mCHG (%)** | **mCHH (%)** | **mCHH (chloroplast)** |
|---|---|---|---|---|---|
| *6b4-1* | 21.3 | 24.6 | 8.3 | 2.3 | 0.5 |
| *6b4-2* | 20.1 | 26.2 | 9.6 | 3.3 | 0.8 |
| *6b4 nac50 nac52* | 19.1 | 28.0 | 8.7 | 3.1 | 0.7 |
| *6b4 jmj14* | 19.8 | 25.5 | 8.1 | 2.2 | 0.5 |
| *6b4 jmj14 cmt3 drm2* | 19.3 | 25.6 | 0.7 | 1.2 | 0.5 |
| *6b4 cmt3 drm2* | 21.3 | 24.9 | 0.8 | 1.4 | 0.6 |
| *6b4 ibm1* | 20.8 | 26.8 | 19.1 | 3.1 | 0.7 |

Note: Mean genomic coverage and methylation percentages for CG, CHG and CHH context. Cytosines covered by 3 or more reads were used to calculate genome-wide methylation percentages.

## Supplementary references


1. Taochy, C. *et al.* A genetic screen for impaired systemic RNAi highlights the crucial role of DICER-LIKE 2. *Plant Physiol* **175**, 1424-1437 (2017).
2. Brosnan, C.A. *et al.* Nuclear gene silencing directs reception of long-distance mRNA silencing in Arabidopsis. *Proc Natl Acad Sci U S A* **104**, 14741-6 (2007).
3. Beclin, C., Boutet, S., Waterhouse, P. & Vaucheret, H. A branched pathway for transgene-induced RNA silencing in plants. *Curr Biol* **12**, 684-8 (2002).
