# Supplementary material for: Contrasting epigenetic control of transgenes and endogenous genes promotes post-transcriptional transgene silencing in Arabidopsis
Source: Nat Commun. 2021 May 13;12:2787. doi: 10.1038/s41467-021-22995-3 (PMC8119426; doi:10.1038/s41467-021-22995-3)
Supplement: Supplementary file 4 — Description of Additional Supplementary Files [file 41467_2021_22995_MOESM4_ESM.pdf]

Description of additional supplementary files

Title: Supplementary Data 1

Description: Summary of Differentially Methylated Regions (DMR) detected in 6b4 jmj14, 6b4 nac50 nac52 and 6b4 ibm1, as compared to 6b4 WT.
