# Supplementary material for: Contrasting epigenetic control of transgenes and endogenous genes promotes post-transcriptional transgene silencing in Arabidopsis
Source: Nat Commun. 2021 May 13;12:2787. doi: 10.1038/s41467-021-22995-3 (PMC8119426; doi:10.1038/s41467-021-22995-3)
Supplement: Supplementary file 5 — Reporting Summary [file 41467_2021_22995_MOESM5_ESM.pdf]

nature research

Corresponding author(s): Vaucheret

Last updated by author(s): 2020, October 5

Reporting Summary

Nature Research wishes to improve the reproducibility of the work that we publish. This form provides structure for consistency and transparency in reporting. For further information on Nature Research policies, see our Editorial Policies and the Editorial Policy Checklist.

Statistics

For all statistical analyses, confirm that the following items are present in the figure legend, table legend, main text, or Methods section.

n/a	Confirmed	
☐	☒	The exact sample size (*n*) for each experimental group/condition, given as a discrete number and unit of measurement
☐	☒	A statement on whether measurements were taken from distinct samples or whether the same sample was measured repeatedly
☐	☒	The statistical test(s) used AND whether they are one- or two-sided<br>*Only common tests should be described solely by name; describe more complex techniques in the Methods section.*
☒	☐	A description of all covariates tested
☒	☐	A description of any assumptions or corrections, such as tests of normality and adjustment for multiple comparisons
☐	☒	A full description of the statistical parameters including central tendency (e.g. means) or other basic estimates (e.g. regression coefficient) AND variation (e.g. standard deviation) or associated estimates of uncertainty (e.g. confidence intervals)
☒	☐	For null hypothesis testing, the test statistic (e.g. *F*, *t*, *r*) with confidence intervals, effect sizes, degrees of freedom and *P* value noted<br>*Give P values as exact values whenever suitable.*
☒	☐	For Bayesian analysis, information on the choice of priors and Markov chain Monte Carlo settings
☒	☐	For hierarchical and complex designs, identification of the appropriate level for tests and full reporting of outcomes
☒	☐	Estimates of effect sizes (e.g. Cohen's *d*, Pearson's *r*), indicating how they were calculated

Our web collection on statistics for biologists contains articles on many of the points above.

Software and code

Policy information about availability of computer code

Data collection	Bisulfite sequencing data was generated by BGI Genomics (Hong Kong).
Data analysis	Bismark_v0.20.0 was used for mapping bisulfite sequencing reads and calculating cytosine methylation levels. All figures presenting bisulfite data were generated using R version 4.0.2 with custom scripts.

For manuscripts utilizing custom algorithms or software that are central to the research but not yet described in published literature, software must be made available to editors and reviewers. We strongly encourage code deposition in a community repository (e.g. GitHub). See the Nature Research guidelines for submitting code & software for further information.

Data

Policy information about availability of data

All manuscripts must include a data availability statement. This statement should provide the following information, where applicable:

- Accession codes, unique identifiers, or web links for publicly available datasets
- A list of figures that have associated raw data
- A description of any restrictions on data availability

Raw data are available on NCBI's Gene Expression Omnibus (GSE152584)

Field-specific reporting

Please select the one below that is the best fit for your research. If you are not sure, read the appropriate sections before making your selection.

☒ Life sciences ☐ Behavioural & social sciences ☐ Ecological, evolutionary & environmental sciences

For a reference copy of the document with all sections, see nature.com/documents/nr-reporting-summary-flat.pdf

Life sciences study design

All studies must disclose on these points even when the disclosure is negative.

Sample size	GUS expression analyses were performed on 96 independent plants of each genotype. Grafting were performed on replicates of at least 16 plants. ChIP were performed on two replicates involving a mix of at least 50 plants. WGBS experiments were performed on a mix of 8 plants.
Data exclusions	no data were excluded
Replication	Biological experiments corresponds to at least two different replicates (i.e. using plants grown independently).
Randomization	not relevant to this study
Blinding	not relevant to this study

Reporting for specific materials, systems and methods

We require information from authors about some types of materials, experimental systems and methods used in many studies. Here, indicate whether each material, system or method listed is relevant to your study. If you are not sure if a list item applies to your research, read the appropriate section before selecting a response.

Materials & experimental systems

n/a	Involved in the study	
☐	☒	Antibodies
☒	☐	Eukaryotic cell lines
☒	☐	Palaeontology and archaeology
☐	☒	Animals and other organisms
☒	☐	Human research participants
☒	☐	Clinical data
☒	☐	Dual use research of concern

Methods

n/a	Involved in the study	
☒	☐	ChIP-seq
☒	☐	Flow cytometry
☒	☐	MRI-based neuroimaging

Antibodies

Antibodies used	anti myc (mouse 9E10, sigma-M4439), anti GFP (Rabbit Abcam-Ab290), anti tubulin (mouse B-5-1-2, sigma-T5168), GFP-Trap Dynabeads (alpaca chromotek-gtd20), anti flag (mouse Sigma-F3165)
Validation	all antibodies are commercialized

Animals and other organisms

Policy information about studies involving animals; ARRIVE guidelines recommended for reporting animal research

Laboratory animals	this study did not involve laboratory animals
Wild animals	this study did not involve wild animals
Field-collected samples	no samples were collected from the fields, all were collected from room chamber and/or greenhouse suitable for GMO cultures
Ethics oversight	no ethical approval was required

Note that full information on the approval of the study protocol must also be provided in the manuscript.
